# SARS-CoV-2 Cross-Reactivity in Prepandemic Serum from Rural Malaria-Infected Persons, Cambodia

**DOI:** 10.3201/eid2805.220404

**Published:** 2022-05

**Authors:** Jillian T. Grassia, Christine F. Markwalter, Wendy P. O’Meara, Steve M. Taylor, Andrew A. Obala

**Keywords:** COVID-19, SARS-CoV-2, coronavirus disease, neutralizing antibodies, cross-reactivity, Plasmodium falciparum, malaria, serosurvey, severe acute respiratory syndrome coronavirus 2, vector-borne infections, viruses, zoonoses, Cambodia, Kenya

**To the Editor:** We read with interest the observations by Manning et al. (*1*) that serum collected from malaria-infected persons in Cambodia before the coronavirus disease (COVID-19) pandemic harbored seroreactivity against severe acute respiratory syndrome coronavirus 2 (SARS-CoV-2) antigens but lacked neutralizing activity. These results suggest that malaria exposure may increase background reactivity in SARS-CoV-2 serosurveys and more specific measures of exposure, such as surrogate virus neutralization tests (sVNTs), may be necessary to capture functional SARS-CoV-2 seroreactivity in malaria-endemic areas. Additional studies in settings with distinct malaria transmission intensities would generalize and strengthen these findings.

One hypothesis for the unexpectedly moderate burden of SARS-CoV-2 in malaria-endemic countries in Africa is that exposure to *Plasmodium falciparum* confers functional protection against COVID-19 through cross-reactivity or general immune activation. To test this hypothesis, we analyzed 237 dried blood spot samples taken in January 2020 (prepandemic) from *P. falciparum*–exposed persons in a high-transmission setting in western Kenya for the presence of SARS-CoV-2 neutralizing antibodies (nAbs) using the GenScript SARS-CoV2 sVNT assay (https://www.genscript.com). Monthly *P. falciparum* real-time PCR results were collected in a previous study (*2*) for 138/237 persons in the 12 months prior to January 2020. Of these, 131 (95%) were infected with *P. falciparum* at least 1 time in 2019, suggesting that most persons included in this screening had been recently exposed to malaria parasites.

Consistent with findings in Manning et al. (*1*), none of the 237 people harbored SARS-CoV-2 nAbs, despite high prior levels of exposure to *P. falciparum.* Although nAbs are subject to decay after infection (*3*), this lack of nAb activity suggests that sVNTs offer a more specific measure of SARS-CoV-2 exposure than standard ELISAs (*4*). We further suggest that, given that protection from SARS-CoV-2 infection may be associated with the presence of nAbs (*5*), their absence in samples from both the Manning et al. study (*1*) and our study does not support the notion that *P. falciparum* infections elicit functional humoral responses against COVID-19.
